# Interaction of Colloidal Gold Nanoparticles with Urine and Saliva Biofluids: An Exploratory Study

**DOI:** 10.3390/nano12244434

**Published:** 2022-12-13

**Authors:** Maria António, Tânia Lima, Rui Vitorino, Ana L. Daniel-da-Silva

**Affiliations:** 1 CICECO-Aveiro Institute of Materials, Department of Chemistry, University of Aveiro, 3810-193 Aveiro, Portugal; 2 iBiMED-Institute of Biomedicine, Department of Medical Sciences, University of Aveiro, 3810-193 Aveiro, Portugal; 3 Cancer Biology and Epigenetics Group, Research Center of Portuguese Oncology Institute of Porto (GEBC CI-IPOP) & Porto Comprehensive Cancer Center (P.CCC), 4200-072 Porto, Portugal; 4 LAQV-REQUIMTE, Department of Chemistry, University of Aveiro, 3810-193 Aveiro, Portugal; 5 UnIC@RISE, Department of Surgery and Phycology, Cardiovascular R&D Center, Faculty of Medicine, University of Porto, 4200-319 Porto, Portugal

**Keywords:** gold nanoparticles, protein adsorption, protein corona, saliva, urine, LC-MS/MS

## Abstract

The use of gold nanoparticles for drug delivery, photothermal or photodynamic therapy, and biosensing enhances the demand for knowledge about the protein corona formed on the surface of nanoparticles. In this study, gold nanospheres (AuNSs), gold nanorods (AuNRs), and gold nanoflowers (AuNFs) were incubated with saliva or urine. After the interaction, the surface of gold nanoparticles was investigated using UV-VIS spectroscopy, zeta potential, and dynamic light scattering. The shifting of the localized surface plasmon resonance (LSPR) band, the increase in hydrodynamic diameter, and the changes in the surface charge of nanoparticles indicated the presence of biomolecules on the surface of AuNSs, AuNRs, and AuNFs. The incubation of AuNFs with saliva led to nanoparticle aggregation and minimal protein adsorption. AuNSs and AuNRs incubated in saliva were analyzed through liquid chromatography with tandem mass spectrometry (LC-MS/MS) to identify the 96 proteins adsorbed on the surface of the gold nanoparticles. Among the 20 most abundant proteins identified, 14 proteins were common in both AuNSs and AuNRs. We hypothesize that the adsorption of these proteins was due to their high sulfur content, allowing for their interaction with gold nanoparticles via the Au-S bond. The presence of distinct proteins on the surface of AuNSs or AuNRs was also investigated and possibly related to the competition between proteins present on the external layers of corona and gold nanoparticle morphology.

## 1. Introduction

Nanoparticles (NPs) are materials with intermediate dimensions between molecules and sub-micrometer particles that exhibit properties distinct from the bulk materials with identical chemical compositions due to their small size. When the average size of the particles diminishes, the surface-to-volume ratio increases [1], which can contribute to the number of loaded biomolecules onto the surface of nanoparticles. Gold, as a metal in the bulk form, presents high electrical conductivity and oxidation resistance [2]. At nanoscale dimensions, gold nanoparticles (AuNPs) exhibit interesting optical properties, namely the localized surface plasmon resonance (LSPR) band in the optical spectrum due to its interaction with light [3]. This LSPR band could shift to a higher or lower wavelength on the optical spectrum depending on the morphology of AuNPs, size, and nature of the surrounding medium [4]. AuNPs, owing to their intrinsic optical, electronic, and physicochemical properties, have been widely investigated for applications in biomedicine [5,6]. Examples of applications include drug delivery [7], photothermal therapy [8,9], photodynamic therapy [10,11], X-ray imaging [12,13], and biosensing [14,15,16]. Additionally, AuNPs exhibit a high surface-to-volume ratio that favors the conjugation with biomolecules [17,18,19,20], are chemically stable, present low toxicity, and are biocompatible [18,21].

AuNPs could be delivered into the bloodstream for drug delivery and photodynamic therapy or could be applied ex vivo for disease diagnosis in serum or plasma. In all these cases, AuNPs will interact with components of the surrounding biological medium [22]. This interaction typically leads to the adsorption of biomolecules, forming a biomolecular corona, also known as a protein corona, that may influence the internalization of AuNPs into cells [23] and affect their biodistribution and cytotoxicity [24]. In clinical assays, this corona may interfere with specific biomolecule detection [25]. Thus, understanding the phenomena that occur on the surface of AuNPs in complex biological environments is essential for further advances in the design of nanomedicines and detection methods [26]. The affinity of human proteins like albumin, fibrinogen, γ-globulins, histone, and insulin to citrate-capped AuNPs has been demonstrated [27,28]. The adsorption of proteins onto the AuNPs’ surface is influenced by the particle size, surface coating, and the pH of the medium [29,30,31]. Further, other works correlated the surface modification of AuNPs with the changes in the conformation of adsorbed proteins [32,33]. In addition, the in vivo protein adsorption in anisotropic AuNPs of different sizes and shapes after blood circulation was investigated [34]. Spherical, flower-like and rod-shaped AuNPs are widely used in biosensing approaches [15,35,36]. Blood, serum, and plasma are biofluids usually used in biomarker research for disease diagnosis. However, their collection is invasive and needs to be done by professionals. Other biofluids, namely urine and saliva, are easier to collect, the collection is non-invasive, and the collection could be done by the patient itself or with help from a relative [37]. Urine has been studied to be applied in the detection of several illnesses [16] such as chronic renal disease [38], Alzheimer’s disease [39], breast cancer [40], cervical cancer [41], and celiac diseases [42]. On the other hand, saliva has been investigated for the identification of childhood autism [43], Alzheimer’s disease [44], oral cancer [45], Cushing’s disease [46], and lung cancer [47]. Despite the recognized utility of urine and saliva, the study of AuNPs’ interaction with saliva and urine components has not yet been reported in the literature.

In this work, AuNPs with distinct morphologies, spherical, flower-like, and rod-shaped, were incubated in urine and saliva samples, and the proteins adsorbed on the surface of AuNPs were identified. The aim of this study is to contribute to a better understanding of the influence of the morphology and coating of AuNPs on the adsorption of biomolecules in biofluids.

## 2. Results

### 2.1. Physicochemical Characterization of AuNPs

**Figure 1:**
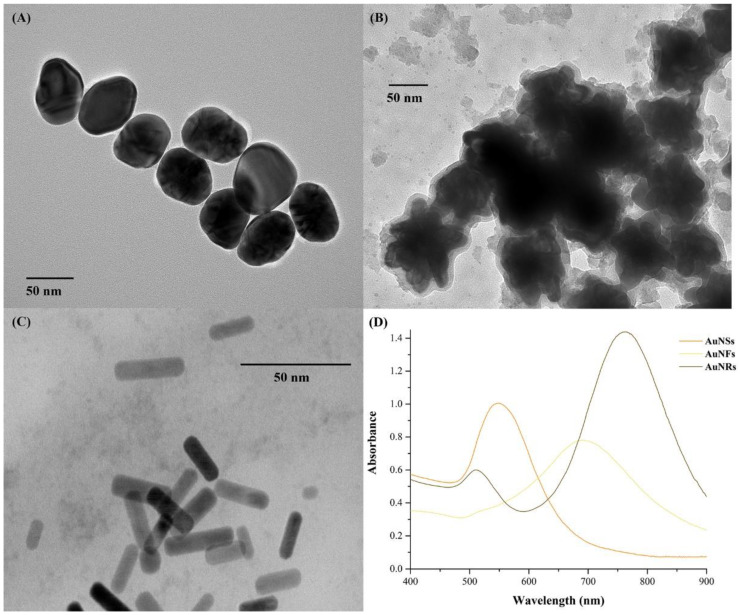
STEM images of (**A**) AuNSs, (**B**) AuNFs, and (**C**) AuNRs, and (**D**) UV-VIS spectra of AuNPs.

The as-synthesized gold nanoparticles were characterized by scanning transmission electron microscopy (STEM). AuNSs exhibit a spheroidal shape with an average size of 55.5 ± 8.2 nm (Figure 1A). AuNFs present a flower/urchin-like shape with an average size of 89.6 ± 15.2 nm (Figure 1B). AuNRs are rod-shaped with an average length and width of 20.5 ± 7.3 nm × 7.1 ± 1.3 nm (Figure 1C), respectively. In Figure 1B,C, it is possible to observe the presence of the capping agent of AuNFs and AuNRs in excess, hydroquinone and CTAB, respectively. The localized surface plasmon resonance (LSPR) band of AuNSs and AuNFs was centered at 549 nm and 645 nm, respectively (Figure 1D). For AuNRs, two bands were observed as expected, at 509 nm (transversal LSPR) and 762 nm (longitudinal LSPR), respectively. The maximum absorbance was 0.632, 0.6837, 0.349 (transversal), and 0.828 (longitudinal) for AuNSs, AuNFs, and AuNRs, respectively. The final concentration of Au was 2.18 × 10^−4^ M for AuNSs, 2.54 × 10^−4^ M for AuNRs, and less than 2.50 × 10^−5^ M for AuNFs, as determined by ICP-MS.

The zeta potential (ZP) of as-synthesized AuNSs and AuNFs was −61.0 ± 0.3 mV (pH 6.3) and −58.1 ± 1.0 mV (pH 3.9), respectively. The negative surface charge of these particles is due to citrate capping. The AuNRs carried a strong positive surface charge (ZP = +71.3 ± 4.9 mV, pH 2.6) owing to the formation of CTAB-based cationic bilayer on their surface [48]. The hydrodynamic diameter (HD) of AuNSs and AuNFs was close to the particle size estimated by electron microscopy (Table S1 of Supplementary Materials). As for AuNRs, the HD was above the primary particle size, as expected based on the large volume of CTAB molecules and the non-spherical shape of the nanoparticles [49]. The values of polydispersity indexes (PDI) were ≤0.3, thus indicating moderate polydisperse size distribution.

### 2.2. Optical Changes of AuNPs after Incubation with Biofluids

Figure S1 of Supplementary Materials shows the optical spectra of the AuNPs before and after incubation with saliva and urine samples. Overall, there was a decrease in the intensity of the bands after incubation. The LSPR band shifted differently over time. Table 1 summarizes the wavelength values of the LSPR band before and after 15, 30, and 60 min of incubation.

The as-synthesized AuNSs presented an LSPR band centered at 549 nm. After 15 min incubation with saliva and urine, the LSPR band red-shifted to 553 and 555 nm, respectively. Longer periods in urine (60 min) led to a blue-shift to 547 nm. The AuNFs initially showed an LSPR band at 645 nm. After incubation with saliva, the band intensity decreased sharply together with a marked red-shift to 750 nm (Figure S1 of Supplementary Materials), suggesting that the AuNFs aggregated. Yet, in the presence of urine, the intensity decreased, and the red-shift of the LSPR band was less pronounced (~12 nm, after 60 min incubation). The transversal LSPR band of AuNRs was also red-shifted after incubation regardless of the biofluid. This shift was more marked in saliva (~7 nm, after 60 min). In contrast, the longitudinal LSPR band, originally at 762 nm, was blue-shifted after incubation. This blue-shift was more pronounced in saliva, after 60 min (~98 nm), while in urine the shift was smaller (~28 nm) but still very relevant. A blue-shift in the longitudinal LSPR of AuNRs after incubation with fetal bovine serum has been reported and ascribed to changes in the dielectric environment due to protein corona formation [50]. The longitudinal LSPR of AuNRs stabilized with CTAB was also blue-shifted in the presence of BSA [51]. This blue-shift was attributed to the interaction of the tryptophan of BSA with the hydrophobic tail of CTAB [52].

Besides the optical properties, the surface charge and hydrodynamic diameter were influenced by the adsorption of biomolecules in saliva and urine.

### 2.3. Effect on Surface Charge and Hydrodynamic Diameter

#### 2.3.1. Incubation with Urine

**Figure 2:**
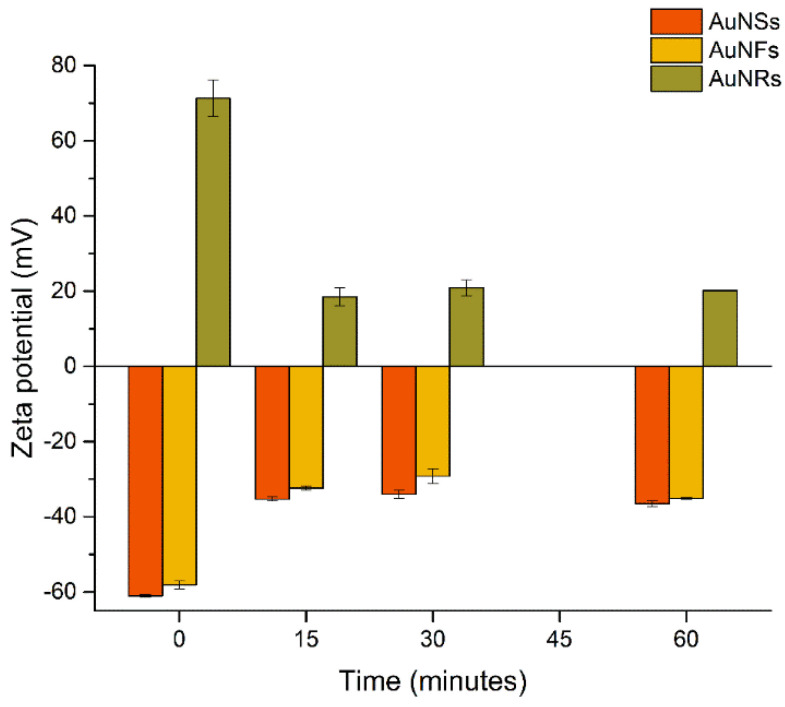
Zeta potential values of AuNPs before and after incubation with urine samples.

Figure 2 shows the zeta potential (ZP) values of AuNSs, AuNFs, and AuNRs before (t = 0 min) and after incubation with urine for 15 to 60 min.

The ZP of incubated AuNSs and AuNFs increased markedly after 15 min from −61.0 ± 0.3 mV (pH 6.3) to −35.3 ± 0.6 mV (pH 5.6) and from −58.1 ± 1.0 mV (pH 3.9) to −32.4 ± 0.6 mV (pH 4.7), respectively. Afterward, only small changes were observed, and after 60 min of incubation, the ZP was −36.6 ± 0.8 mV (pH 5.5) and −35.1 ± 0.2 mV (pH 4.9) for AuNSs and AuNFs, respectively. The lower negative surface charge suggests the adsorption of positively charged biomolecules or cations such as Na^+^, K^+^, and Ca^2+^ in urine [53]. Regarding AuNRs, after 15 min of incubation the ZP sharply decreased from +71.3 ± 4.9 mV (pH 2.6) to +18.5 ± 2.4 mV (pH 2.1) and remained invariable for longer periods, being +20.2 ± 0.1 mV (pH 2.1) after 60 min incubation. In this case, a less positive charge could indicate the adsorption of negatively charged biomolecules or anions such as Cl^−^ in urine [53].

**Figure 3:**
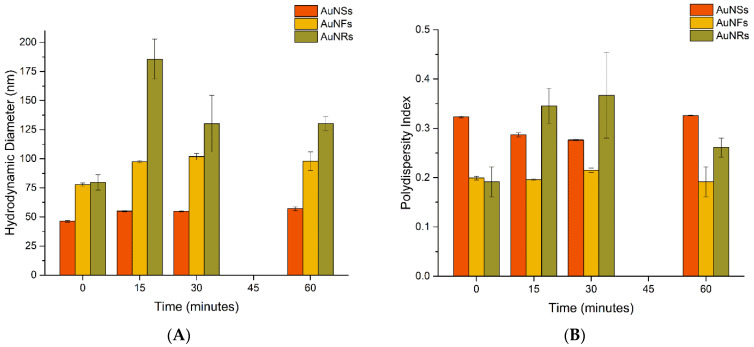
(**A**) Hydrodynamic diameter and (**B**) polydispersity index of AuNPs before and after incubation with urine samples.

Figure 3A shows the variation of hydrodynamic diameter (HD) and Figure 3B shows PDI of AuNPs over time after incubation in urine.

The HD of AuNSs slightly increased from 46.3 ± 0.7 nm (as-synthesized) to 54.9 ± 0.5 nm after 15 min and then up to 57.1 ± 1.7 nm after 60 min of incubation. The PDI values of AuNSs after incubation decreased from 0.323 ± 0.002 to 0.286 ± 0.004 after 15 min and increased to 0.326 ± 0.001 after 60 min of incubation. For AuNFs, the increase of HD was more expressive, from 77.9 ± 1.3 nm (as-synthesized) to 97.5 ± 1.0 nm after 15 min of incubation. No marked changes were observed afterward, being 97.9 ± 2.1 nm after 60 min. The PDI slightly varied in the range 0.192–0.215, indicating a moderately polydisperse size distribution. The HD of AuNRs before incubation was 79.6 ± 6.6 nm, which increased after 15 min of incubation to 185.5 ± 17.2 nm and decreased at 30 min to 130.2 ± 24.5 nm. At 60 min, the HD value remained similar 130 ± 6.2 nm. Significant alterations in the PDI values of AuNRs were noticed after incubation with urine. The PDI values considerably increased to 0.345 ± 0.036 and 0.367 ± 0.087 for incubation times of 15 and 30 min, when compared with the PDI value before incubation of 0.192 ± 0.030. At 60 min of incubation, the PDI decreased to 0.261 ± 0.019. As could be noticed, the standard deviation of AuNRs was higher than the other nanoparticles, which could be related to the rod-shaped morphology and the use of the DLS technique [54].

#### 2.3.2. Incubation with Saliva

**Figure 4:**
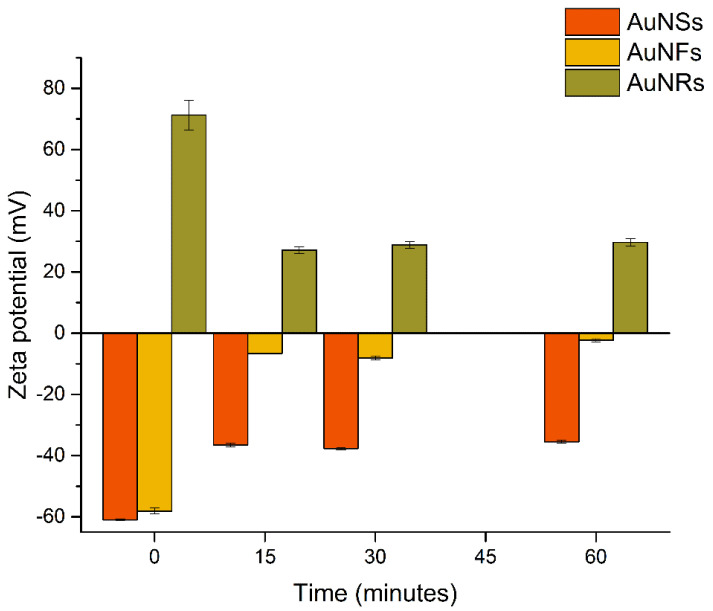
Zeta potential values of AuNPs before and after incubation with saliva samples.

**Figure 5:**
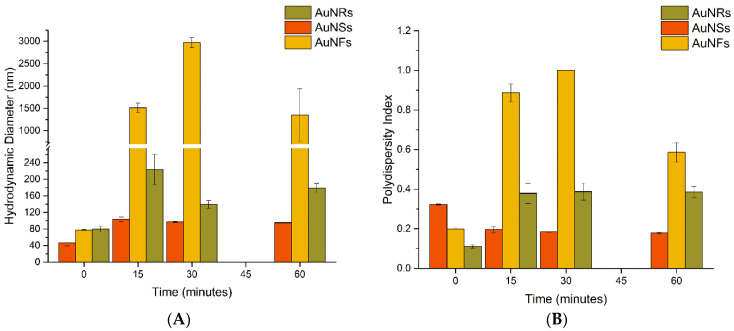
(**A**) Hydrodynamic diameter and (**B**) polydispersity index of AuNPs before and after incubation with saliva samples.

Figure 4 shows the ZP results, and Figure 5 shows the (A) HD and (B) PDI values resulting from incubating AuNPs with saliva.

When incubated in saliva, the surface charge of AuNSs and AuNFs increased (less negative ZP value), similar to what was observed for urine incubation. The ZP values of AuNSs and AuNFs increased from −61.0 ± 0.3 mV (pH 6.3) and −58.1 ± 1.0 mV (pH 3.9) (before incubation) to −35.5 ± 0.5 mV (pH 6.4) and −2.3 ± 0.5 mV (pH 3.6), respectively, after 60 min of incubation. AuNRs showed a significant decrease in ZP values from +71.3 ± 4.9 mV (pH 2.6) to 27.2 ± 1.1 mV (pH 2.2) after 15 min, keeping unchanged over time (after 60 min, 29.7 ± 1.2 mV, pH 1.9). Despite the decrease in ZP, both AuNSs and AuNRs seem to present colloidal stability in saliva (|ZP| ≥ 30 mV) [49]. In contrast, the low value of ZP for AuNFs is indicative of colloidal instability.

The HD of AuNSs increased from 46.3 ± 6.7 nm to 103.7 ± 5.4 nm after 15 min and then stabilized at around 97.3 ± 1.7 nm after 30 min. The PDI decreased from 0.323 ± 0.002 (as-synthesized) to values lower than 0.2 after incubation. The size increase in AuNRs was more marked. Overall, the HD of AuNRs increased after incubation, reaching the highest value after 15 min of incubation (223.1 ± 37.0 nm) and then decreased to 178.8 ± 11.0 nm after 60 min. The PDI of AuNRs also increased gradually from 0.11 ± 0.008 before incubation to 0.386 ± 0.027 after 60 min incubation, revealing an increase in the aggregation tendency of the AuNRs in saliva. Regarding AuNFs, a very sharp increase of HD was observed, from 77.9 ± 1.3 nm to 2973.5 ± 111.5 nm and 1348.6 ± 588.4 nm, after 30 and 60 min of incubation, respectively. This huge increase in the HD of AuNFs suggested the aggregation of the nanoparticles, which is in agreement with the variation of optical properties described above. Additionally, the PDI analysis shows an increase from 0.199 ± 0.004 before incubation to 0.886 ± 0.046 and 1.00 ± 0.00 after 15 and 30 min incubation, respectively (Table S2, Supplementary Materials), which is in line with the aggregation of AuNFs.

To assess the proteins adsorbed on the surface of the nanoparticles, an SDS-PAGE analysis followed by an LC-MS/MS analysis was completed.

### 2.4. Protein Analysis

**Figure 6:**
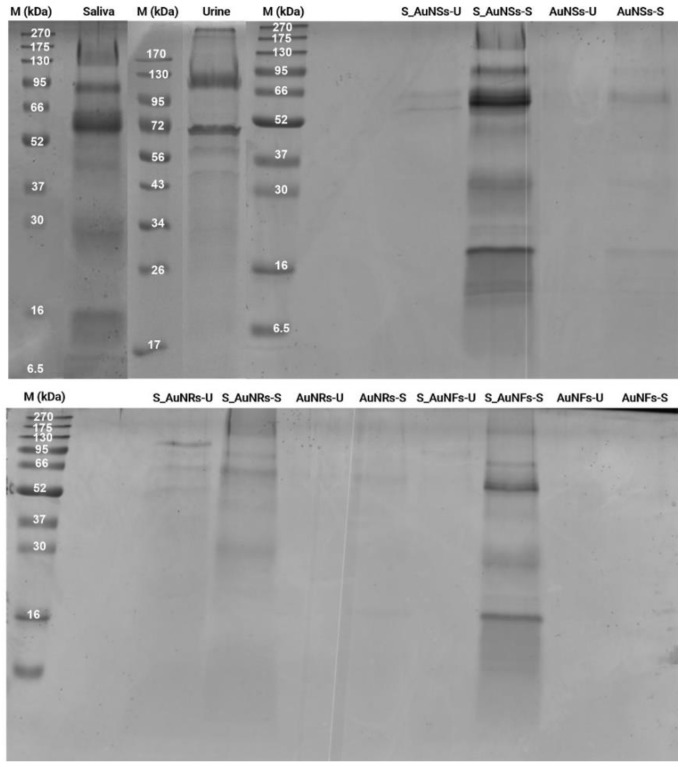
SDS-PAGE (12%) gel achieved after the incubation of AuNSs, AuNRs, and AuNFs incubated with urine (U) or saliva (S). M: Marker; Control: Diluted saliva used as reference; S: Supernatant of AuNPs.

SDS-PAGE was performed to assess the proteins in the supernatant and adsorbed on the surface of the nanoparticles (Figure 6).

All AuNPs tested did not adsorb any significant amount of protein after incubation with urine. Additionally, the presence of protein in the supernatant recovered after incubation of AuNPs with urine was low, which was expected since urine has a low protein content [55]. Regarding AuNSs incubated in saliva (AuNSs-S), proteins with a molecular weight of about 95 kDa, 60 kDa, 40 kDa, 30 kDa, and 20 kDa were adsorbed onto the surface of the nanoparticles. In the case of AuNRs (AuNRs-S), proteins with a molecular weight of about 175 kDa, 60 kDa, and 16 kDa were adsorbed onto the surface of the nanoparticles. The pattern of the supernatant (S_AuNRs-S) was similar to the control but with less protein than AuNSs-S. The AuNFs did not show evidence of protein adsorption after incubation with saliva (AuNFs-S), which can be related to their aggregation during the experiment. The HD results (Figure 5) indicate the formation of large aggregates of AuNFs that are expected to have less contact area with the surrounding biofluids, leading to less protein adsorption. The supernatant profile (S_AuNFs-S) was similar to the control, as observed in Figure 6.

The presence of proteins on the surface of AuNPs after incubation with urine was minimal. In contrast, after incubation with saliva, adsorbed proteins could be observed on the surface of AuNPs (Figure 6). The proteins adsorbed onto AuNSs and AuNRs incubated in saliva were analyzed by LC-MS/MS. The pattern of bands was different, suggesting the presence of distinct adsorbed proteins on the surface of AuNSs and AuNRs.

**Figure 7:**
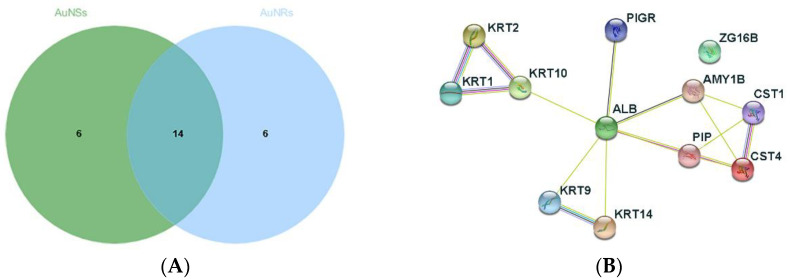
(**A**) The 20 most abundant proteins on the surface of AuNSs and AuNRs; (**B**) corresponding PPI network.

For the LC-MS/MS study, we considered only identified master proteins with at least two peptides. Given this pre-selection, a total of 114 proteins were identified, and of these, 10 were found to play a significant role in disease diagnosis. These proteins are the cystatin C and D, neutrophil gelatinase-associated lipocalin (NGAL), cathepsin B and D, serpin B3, alpha-1-antitrypsin, metalloproteinase inhibitor 1, zinc-alpha-2-glycoprotein, galectin-3 binding protein, SPARC-like protein 1, and complement C3. From the pre-selected proteins, 96 were found on the surface of both AuNSs and AuNRs (Table S3, Supplementary Materials). The interactions of these 96 proteins were investigated using String [56] (Figure S2 of Supplementary Materials). The found network revealed 73 proteins connected by 347 edges, while only 35 edges were expected. Furthermore, the protein–protein interaction (PPI) enrichment *p*-value was lower than 1.0 × 10^−16^ (Figure S2 of Supplementary Materials). These proteins are associated with molecular functions such as endopeptidase inhibitor activity, protease binding, structural constituent of the cytoskeleton, and enzyme regulator activity with a *p*-value lower than 5.0 × 10^−4^. According to the Human Salivary Proteome Wiki database, only 56 out of 96 proteins have been identified in the oral mucosa or salivary gland [57]. The PPI network of the 56 proteins is shown in Figure S3 of the Supplementary Materials. The network revealed 52 proteins connected by 161 edges while only 10 edges were expected. Likewise, the PPI enrichment *p*-value was found to be less than 1.0 × 10^−16^. The molecular function remains similar to the described for the 96 proteins but focused on endopeptidase inhibitor activity and protease binding with a *p*-value of less than 5.0 × 10^−5^. The 20 most abundant proteins on the surface of AuNSs and AuNRs are presented in Table S4 of Supplementary Materials, and 14 common proteins were observed to exist (Figure 7B). The PPI network (Figure 7A) shows 12 proteins connected through 16 edges with 1 being expected, with a PPI enrichment *p*-value lower than 1.0 × 10^−16^. The 40 identified proteins were investigated by comparing their molecular weight, isoelectric point (pI), and gravy value [58] (Table S4 of Supplementary Materials). The proteins had different molecular weights, ranging from 11.8 to 83.2 kDa, a pI varying from 5.02 to 9.16, and negative gravy values ranging from −0.700 to 0.175.

The 14 proteins adsorbed on the surface of both AuNSs and AuNRs have molecular weights ranging from 11.8 to 69.3, a pI ranging from 5.02 to 8.12, and gravy values varying from −0.700 to 0.175. Those proteins were the keratin type I cytoskeletal 9 (KRT9), 10 (KRT10), and 14 (KRT14); keratin II cytoskeletal 1 (KRT1) and 2 epidermal (KRT1); α-amylase 1 (AMY1B); albumin (ALB); cystatin-S (CST4); cystatin-SN (CST1); prolactin-inducible protein (PIP); zymogen granule protein 16 (ZP16B); polymeric immunoglobulin receptor (PIGR); and immunoglobulins heavy constant α and ᴋ constant. The adsorption of albumin on citrate-capped AuNSs [29] and CTAB-capped AuNRs [59] has been reported and is ascribed to Au-S interaction. Furthermore, the interaction between AuNSs and α-amylase was already observed [60], as well as the interaction between AuNSs and IgM and IgG. The IgG directly interacts with the metal surface and causes citrate displacement [61]. The adsorption of keratins could be explained by their higher molecular weight (51.5–66 kDa) [22]. The proteins α-amylase, immunoglobulins heavy constant α, prolactin-inducible protein, and polymeric immunoglobulin receptor contain in their structure 12, 15, 5, and 21 cysteines of a total of 511, 353, 146, and 764 amino acids, respectively. The cysteine has a thiol group that provides affinity for Au surface and could explain the presence of these proteins on the surface of AuNSs and AuNRs (Table S4 of Supplementary Materials). As a reference, albumin has 30 cysteines of 606 amino acids. However, the presence of zymogen granule protein 16 is difficult to justify as no cysteines are present and have medium molecular weight.

Ten proteins were exclusively identified on the surface of AuNSs and eight proteins on the surface of AuNRs. The AuNSs and AuNRs differ in morphology—spherical versus rod-like shape, and in the stabilizing agent—citrate versus CTAB, respectively. Citrate and CTAB affinity to proteins was investigated using STITCH [62] and SwissTargetPrediction [63]. For citrate, results were not found using STITCH. However, using SwissTargetPrediction, 64 proteins were found (Table S5 of Supplementary Materials) but were different from those identified in this study (Table S3 of Supplementary Materials). In the case of CTAB, the molecule does not match the STITCH molecules database, and when using SwissTargetPrediction, an error was found in the database research. Since no conclusions were found, the 18 proteins were investigated by comparing the molecular weight, isoelectric point (pI), gravy value [58], and abundance (Table S6 of Supplementary Materials). The proteins had different molecular weights, from 13.1 to 161 kDa, a pI varying from 5.17 to 9.29, and negative gravy values ranging from −0.00513 to −0.983. Proteins absorbed by AuNSs exhibited a wider range of molecular weights (13.1–161 kDa) than proteins absorbed by AuNRs (29.5–121.3 kDa). In addition, the proteins absorbed by AuNRs had more restricted pI values (5.17–7.08) and gravy values (−0.151 to −0.983) if compared to proteins adsorbed by AuNSs. Negatively charged proteins were expected to be adsorbed on the surface of positively charged AuNRs and positively charged proteins on the surface of negatively charged AuNSs. At pH ~6, AuNSs were negatively charged, and the proteins positively charged, i.e., proteins having pI > 6, were able to interact electrostatically with AuNSs, as listed in Table S4 of the Supplementary Materials. However, some proteins with pI values between 5 and 6 also adsorbed to the AuNSs surface. Moreover, positively charged AuNRs were observed to interact with proteins with a net positive charge, at pH ~2 [64] (Table S6 of Supplementary Materials). Since the usual pI of proteins is higher than 4, the electrostatic interactions were unfavorable in the case of AuNRs. However, because of their complex structure, proteins may exhibit different affinities and local charges, depending on the local composition of amino acid residues. Proteins adsorbing at charged interfaces tend to expose oppositely charged regions to the surface [65]. Further, ionizable groups of proteins may have a different charge at the particle surface than in bulk phase due to the influence of the local electrostatic environment, a phenomenon known as the charge regulation effect [66]. These two effects combined explain the frequent experimental finding that charged proteins can adsorb to a like-charged surface [67,68]. As the incubation time was 1 h, there may have been several changes on the nanoparticle surface during this period because protein adsorption is a dynamic process with proteins continually adsorbing and desorbing with time [26]. Proteins with low affinity are expected to loosely adsorb on the surface of AuNPs for a short time and dynamically interchange with the surrounding medium, forming the so-called “soft corona”. In contrast, proteins with high affinity for the particle surface form a long-term layer known as “hard corona” [69]. After 1 h, the proteins that are on the surface of AuNPs, and the contributions of van der Waals and protein–protein interaction determine the composition of the protein corona [70].

## 3. Discussions

In this work, we investigated the changes in the physicochemical properties of gold nanoparticles with distinct morphology after incubation in urine and saliva samples. The AuNPs bearing negative surface charge, AuNSs and AuNFs, showed an identical ZP increase (less negative values) after incubation in urine. In saliva, this variation was more marked in AuNFs. The increase in ZP values indicated the adsorption of biomolecules or ionic species present in the biofluids. For the positively surface-charged AuNRs, the ZP value decreased after incubation in the biofluids, also suggesting the adsorption of species surrounding. Because AuNRs display an opposite surface charge than AuNSs and AuNFs, the biomolecules adsorbed onto the surface of the nanoparticles should have different characteristics, such as conformation, molecular weight, and isoelectric point (pI). Proteins with a different pI carry different charges in a given solution that will influence the electrostatic interactions with the surface of the particles [71,72]. Regarding the HD variation, all the AuNPs showed an increase in HD values after being in contact with saliva and urine samples. This variation is ascribed to the adsorption of biomolecules onto the surface of the nanoparticles. However, the composition of saliva and urine biofluids is very different. Since urine has low protein content [55], we can assume that AuNPs interacted with other molecules present in urine, such as creatine, urea, and uric acid [53,73]. The intrinsic charge of these biomolecules depends on the pH of the medium. These molecules have different pKa, which allows for their interaction with differently charged nanoparticles depending on the pH of the medium. Uric acid has a pKa of 5.6; thus, at physiological pH (7.4), it is negatively charged [74]. Creatinine, on the other hand, has a pKa of 4.8 and is positively charged at a pH of 2–5 [75], so creatinine can interact with AuNFs. In addition, urea can undergo protonation [76] and establish electrostatic interactions with AuNPs. Other chemical interactions, such as the formation of the strong Au-S bond, must be accounted for in the interaction of AuNPs with biomolecules [77]. For example, parvalbumin and ovalbumin are low molecular weight proteins present in urine [78] with amino acids containing sulfur that makes them suitable to interact with AuNPs.

In saliva, the maximum HD of both AuNSs and AuNRs was observed after 15 min, which should correspond to maximum protein adsorption. After, a slight decrease was observed, which is consistent with a dynamic interchange of proteins with the surrounding medium in the formation of soft and hard corona [79]. Of significance, AuNSs show colloidal stability after incubation with saliva, supported by |ZP| ≥ 30 mV and PDI values below 0.2, which could be ascribed to the interaction of proteins such as albumin with the citrate-coated gold nanoparticles [15]. Compared with spherical AuNPs, the AuNFs presented a larger surface area due to numerous tips [80]. Therefore, AuNFs were expected to be more prone to bind to proteins than AuNSs due to their small surface curvature (larger size) [69], which contributes to the formation of a hard corona [29]. Additionally, the interaction of proteins such as immunoglobulin G (IgG) with AuNPs could lead to their aggregation, as these proteins can cross-link with citrate-capped AuNPs [61], which could help to explain the aggregation of AuNFs. The presence of hydroquinone on the surface of AuNFs could also contribute to the aggregation of AuNFs, as the carbonic anhydrase II, III, and XII were found to be possible targets of hydroquinone in agreement with the SwissTargetPrediction [63]. These enzymes are present in saliva, according to the Human Salivary Proteome Wiki database [57].

We assume that most of the adsorption is due to the interaction of proteins or ions with the surface of AuNPs. As reported, the formation of protein corona depends on the surface area, hydrophilicity, morphology, and functional groups present on the surface of the nanoparticles, in addition to the physicochemical properties of proteins [81]. This interaction often referred to as adsorption, can be attributed to electrostatic interactions, hydrophobic interactions, van der Waals’ forces, solvation forces, and hydrogen bonding [79]. Some studies revealed the affinity of lysine, arginine, serine, and threonine for citrate ions on the surface of AuNPs [71,82]. It was also found that the first proteins adsorbed on the surface of AuNPs are present in high concentrations and have high association rate constants. However, over time, these proteins are replaced by others that have a high affinity for the surface of AuNPs and are present at lower concentrations [83,84]. Previously, it was observed that AuNSs and AuNRs can adsorb the same proteins, but it was also found that proteins are exclusively present on the surface of AuNRs or AuNSs. The proteins that interact with AuNPs via the Au-S bond must be located on the first of the multiple layers that form the protein corona [79]. Indeed, we have demonstrated the presence of protein-rich proteins, such as albumin, α-amylase, immunoglobulins’ heavy constant α, prolactin-inducible protein, and polymeric immunoglobulin receptor, on the surface of AuNSs and AuNRs. Recent literature supports the presence of albumin on surface of AuNRs modified with PEG in vivo mice [34] and that albumin is replaced after long periods of incubation (h) [85]. Furthermore, protein competition is present in protein corona [70], and the amount of each protein in the first layers of the corona is different for AuNSs and AuNRs, which may contribute to the subsequent adsorption of different proteins. In addition, the abundance of common proteins adsorbed on AuNSs and AuNRs was about 10^3^ higher than the proteins absorbed only by the AuNSs or AuNRs (Table S4 of Supplementary Materials). The adsorption of different proteins by AuNSs or AuNRs could be possibly related to the morphology of the AuNPs, as this may affect the adsorption of proteins to the outer layer of the protein corona.

The results of this study suggest that AuNRs may be more suitable for clinical applications when compared to AuNFs and AuNSs, with respect to protein adsorption. AuNFs aggregate when incubated with saliva. The AuNSs presented a relative abundance of the 20 most abundant proteins higher than the AuNRs (Table S4, Supplementary Materials). The relative abundance of 13 proteins on the surface of AuNSs was higher than 1 × 10^8^, while on the surface of AuNRs, only 7 proteins present similar relative abundance values. Nevertheless, there are several concerns in the clinical translation of AuNRs due to the toxicity of CTAB in cells, and their clinical application requires more investigation [86].

## 4. Materials and Methods

### 4.1. Materials

The chemicals tetrachloroauric (III) acid trihydrate (HAuCl_4_·3H_2_O, ≥99.9%), hydroquinone (C_6_H_6_O_2_, >99%), sodium citrate tribasic dihydrate (Na_3_C_6_H_5_O_7_·2H_2_O, ≥99.0%), hexadecyltrimethylammonium bromide, CTAB (C_19_H_42_BrN, 99%), silver nitrate (AgNO_3_), and sodium borohydride (NaBH_4_, 95%) were purchased from Sigma-Aldrich (Burlington, MA, USA). The hydrochloric acid (HCl, 37%) and formic acid (CH_2_O_2_, 98%) was purchased from Fluka (St. Gallen, Germany), and ascorbic acid (C_6_H_8_O_6_) was acquired from Carlo Erba (Val-de-Reuil, France). Ammonium bicarbonate (NH_4_HCO_3_, ≥97.5%), iodoacetamide (ICH_2_CONH_2_, ≥98%), acetonitrile (H_3_CCN, ≥99.5%), and 1,4-dithiothreitol (C_4_H_10_O_2_S_2_, ≥98%) were purchased from VWR (Pennsylvania, USA). The modified porcine trypsin was provided from Thermo Fischer Scientific (Waltham, MA, USA). There was no further purification of the chemicals acquired.

### 4.2. Synthesis of Gold Nanospheres

The spherical gold nanoparticles (AuNSs) were synthesized via the seed-growth method according to Lekeufack et al. [87]. Gold seeds (with an average size of 15 nm) were firstly prepared using a procedure based on the Turkevich method. In a round-bottom flask of 250 mL, 45 mL of Milli-Q water was brought to boil under reflux. Then, 4.99 mL of 10 mM HAuCl_4_ was added under stirring (300 rpm). Afterward, 5.09 mL of 39.50 mM sodium citrate solution was added, and the solution was left to react until the color was red-wine. The next stage consisted of the growth of the Au seeds that were previously prepared. Thus, 121.89 mL of Milli-Q water was brought to boil under reflux in a round-bottom flask of 250 mL. Next, 3.12 mL of 10 mM HAuCl_4_ was added under reflux and stirred (300 rpm) at 85 °C. Afterwards, 1.13 mL of the previously prepared Au seeds (15 nm) was added, followed by the addition of 0.57 mL of 39.50 mM sodium citrate solution. The solution color changed from purple to pink. After reacting for 30 min, 5.09 mL of 39.50 mM sodium citrate solution was added. Then, it was left to react for 1 h further.

### 4.3. Synthesis of Gold Nanoflowers

Gold nanoflowers (AuNFs) were produced via the seed-growth method, as previously reported [80]. Briefly, the seeds were synthesized by adding 2.7 mL of 1% sodium citrate solution to 100 mL of 0.01% chloroauric solution, with constant stirring and under boiling conditions. The growth solution was prepared by adding 0.75 mL of 1% chloroauric solution to 100 mL of Milli-Q water under vigorous stirring at room temperature. Then, 0.5 mL of the gold seeds, 220 µL of 1% sodium citrate, and 1 mL of 0.03 M hydroquinone were added sequentially to the growth solution. The solution was kept reacting for more than 30 min at room temperature with constant stirring to form gold nanoflowers.

### 4.4. Synthesis of Gold Nanorods

Rod-shaped gold nanoparticles (AuNRs) were synthesized using a seedless method adapted with slight modifications from the literature [88]. HAuCl_4_ (14.25 mL, 1 mM) was added to CTAB (14.25 mL, 0.2 M), followed by the addition of 750 µL of 4.0 mM of AgNO_3_ solution. The resulting solution was gently shaken by inverting the tube once. Then, 24 µL of a 37% HCl solution was added to the solution, followed by 210 µL of 78.8 mM ascorbic acid. The tube was gently inverted several times until the solution became clear, and finally, 45 µL of ice-cold 10 mM NaBH_4_ solution was added. The tube with the resulting solution was inverted once. The mixture was allowed to react without stirring for 3.5 h at 26.3 °C.

### 4.5. Collection of Urine and Saliva Samples

The saliva and urine samples were collected and pre-treated before being used. Urine was collected as the first urine of the morning in a falcon tube (15 mL) and kept in an ice bath until centrifugation. The urine was centrifuged at 3000× *g* for 10 min at 4 °C. The supernatant was recovered and separated to Eppendorf’s with equal sample volume (100 µL) and frozen at −20 °C before use. Then, the urinary samples were defrosted and kept in an ice bath during the trials. Saliva was collected one hour after breakfast and teeth brushing. The sample was collected to an Eppendorf (at least 1 mL of the sample) and placed in an ice bath. Then, saliva was centrifuged at 12,000× *g* for 20 min at 4 °C. The cleared supernatant was separated from the remaining pellet, placed into Eppendorf’s with equal volume (100 µL), and frozen at −20 °C until use.

### 4.6. Incubation of Gold Nanoparticles with Biofluids

Gold nanoparticles with distinct morphologies (AuNSs, AuNFs, and AuNRs) were placed in contact with saliva or urine. In a typical trial, 30 µL of undiluted saliva or urine was added to 200 µL of AuNSs, AuNFs, and AuNRs and incubated until 1 h at 25 °C under stirring (100 rpm). The effect of time of contact was investigated for 15, 30, and 60 min. In the end, the sample was analyzed through UV-VIS spectroscopy (Thermo Fisher Scientific Inc., Waltham, MA, USA). Then, 800 µL of M.Q. water was added to 200 µL of incubated AuNPs and the pH, zeta potential, and hydrodynamic diameter were measured.

### 4.7. Protein Separation through the SDS-PAGE Technique

To investigate the capability of AuNSs, AuNFs, and AuNRs to adsorb proteins, a sodium dodecyl sulfate 12% polyacrylamide gel electrophoresis (SDS-PAGE) was performed. After incubation of Au nanoparticles with biofluids, the AuNSs and AuNFs were centrifuged at 8500 rpm for 20 min, and the AuNRs were centrifuged at 13,300 rpm for 15 min. Then, the supernatant and the centrifuged nanoparticles were placed at Eppendorf microtubes and kept at −20 °C until SDS-PAGE analysis. The nanoparticles and respective supernatant were defrosted and then boiled in loading buffer (400 µL 10% SDS, 600 µL Tris-HCl 0.5M pH 6.8) to ensure protein denaturation and confer an overall negative charge. The resulting solution was applied to the gel for protein separation by electrophoresis (120 V) until completion. After the running, the gel was left overnight in a solution of methanol (40%) and acetic acid (10%) for fixation and lately stained using a colloidal Coomassie solution (2 days). Finally, a methanol solution (25%) was used to unstained the gel.

### 4.8. LC-MS/MS, Protein Identification, and Quantitation

#### 4.8.1. Gels Preparation for LC-MS/MS

The protein bands were excised manually from gels and set into Eppendorf microtubes. The tryptic digestion was completed by applying the modified method [89]. In brief, gel pieces were washed using 25 mM ammonium bicarbonate, ammonium bicarbonate in 50% acetonitrile, and acetonitrile. The cysteine residues were reduced using 10 mM 1,4-dithiothreitol and alkylated with 55 mM iodoacetamide. This process was repeated, and the gel pieces were dried using a SpeedVac. Gel pieces were then hydrated in a digestion buffer with modified porcine trypsin (1:30 (*w*/*w*) substrate ratio in 50 mM ammonium bicarbonate). The gel pieces were incubated in ice for 30 min, and 50 μL of 50 mM ammonium bicarbonate was added to the resulting pellet. Samples were incubated overnight (37 °C), and the tryptic peptides were extracted through the addition of 5% formic acid, and then 5% formic acid and 50% acetonitrile. Finally, the resulted peptides were lyophilized in SpeedVac and resuspended in a 1% formic acid solution.

#### 4.8.2. LC-MS/MS Analysis of the Protein

The proteins adsorbed on the surface of AuNPs were analyzed by LC-MS/MS. The resulted peptides were trapped in 96% solvent A comprising deionized water with 0.1% formic acid at 30 μL/min. The elution was completed using solvent B comprising formic acid and acetonitrile, with a 0.1:80 (*V/V*) ratio at 300 nL/min. The 92 min gradient was completed as described: from 0 to 3 min utilizing 96% solvent A, from 3 to 70 min utilizing 4–25% solvent B, from 70 to 90 min utilizing 25–40% solvent B, from 90 to 92 min utilizing 90% solvent B, from 90 to 100 min utilizing 90% solvent B, and from 101 to 120 min utilizing 96% solvent A. When finishing the LC, the MS analysis was performed in data-dependent acquisition mode at 1.8 kV. The MS2 method was applied with an FT survey scan from 400–1600 *m*/*z*, a resolution of 70,000, and AGC target 1E6. Only the 10 most intense peaks were subjected to HCD fragmentation with a resolution of 17,500, AGC target 5E4, NCE 28%, and 100 ms of max injection time with a dynamic exclusion of 35 s.

For identification and label-free quantification of peptides, the MaxQuant (version 1.6.5.0) software packages were utilized. The MS /MS spectra were examined against the UniprotKB/Swiss-Prot protein sequence database under Homo sapiens (December 2018 version) utilizing Andromeda. The variable modifications chosen were phosphorylation, protein N-term acetylation, and methionine oxidation, as a fixed modification the carbamidomethylation of cysteine was preferred. For MaxQuant, the mass tolerance for the precursor mass and fragments were 20 ppm and 0.15 Da, respectively. It was defined that the lowest peptide length was of 7 amino acids, and the highest of 2 failed cleavages. The FDR for identification was decided to be 1% at peptide and protein levels.

### 4.9. Localization, Properties, and Interaction of Proteins

#### 4.9.1. Venn Diagram Construction

The jvenn was applied to produce Venn diagrams accessible at http://jvenn.toulouse.inra.fr/app/index.html (accessed 4 January 2022). Venn diagrams were employed to distinguish intersections of proteins identified on the surface of AuNSs and AuNRs [90].

#### 4.9.2. String

The String is used to investigate direct and indirect interactions between predicted and known proteins [56]. In this work, String was applied to explore the interactions between proteins adsorbed on the surface of AuNSs and AuNRs. The Uniprot ID of these proteins was placed into the “multiple proteins” box as “P15515, Q96DR5, P01033, P09211, and P01876” (as an example), the host organism selected was “Homo sapiens”, no other options were modified, and the “Search” button was pressed. Then, the option “Continue” was chosen and the protein–protein interaction network was generated; then, the “Legend” and “Analysis” were analyzed to better understand the results. This bioinformatic tool did not recognize most immunoglobulins identified by LC-MS/MS.

#### 4.9.3. The Human Salivary Proteome Wiki

The Human Salivary Proteome Wiki: A Community-Driven Research Platform was created as a public database to assemble all the knowledge about the salivary proteome [57]. This platform is available at https://www.salivaryproteome.org/public/index.php/Main_Page (accessed on 4 January 2022). In the present work, the uniport code of selected proteins present on the surface of AuNSs and AuNRs, or possibly on the surface of AuNFs, was added to the “Search this wiki” and assessed in the topic “Expression” where the existence of each protein in the “Oral mucosa” or “Salivary gland” at the “Protein Localization (score)” was confirmed. Some immunoglobulins were not recognized and did not reproduce any results in the database.

#### 4.9.4. Gravy Calculator

The gravy calculator was created to define the hydrophobicity index of amino acid sequences using the method established by Kyte and Doolittle [58]. The calculator is available at http://www.gravy-calculator.de/ (acessed on 4 January 2022). In this work, the FASTA sequence of each selected protein present on the surface of AuNSs and AuNRs was positioned at the “Input” box, and the latest options were left as default. UniProt was used to obtain the FASTA sequence of each protein. For that, the UniProt ID of each protein was put at “Provide your identifiers” box and the latest options were left as default. Simply “Reviewed” proteins were considered and downloaded using the option “Download” followed by “Uncompressed” to get the FASTA sequences of the proteins. The obtained sequences were used for the gravy calculator.

#### 4.9.5. STITCH Search Tool for the Interaction of Chemicals

STITCH is a bioinformatic tool that combines information about the interaction between proteins and small molecules [62]. It can be accessed through the website: http://stitch.embl.de/cgi/input.pl?UserId=mz0bCVwlVu6D&sessionId=V6mIp6pJH2RJat (accessed on 4 January 2022). In this work, STITCH was used to identify proteins that are known to interact with citrate or CTAB molecules: To do that, the SMILES sequence of citrate and CTAB were positioned into the “multiple chemicals by structures” box, and the “organism” selected was Homo Sapiens. After that, the search button was pressed, and several structures were presented. The SMILES sequence of citrate and CTAB were achieved using the PubChem database, available at https://pubchem.ncbi.nlm.nih.gov/ (accessed on 4 January 2022).

#### 4.9.6. SwissTargetPrediction

The SwissTargetPrediction tool was created to present the most probable protein targets of a small molecule [63], accessed online through http://www.swisstargetprediction.ch/ (accessed on 4 January 2022). As in STITCH, SwissTargetPrediction was used to identify proteins that can interact with citrate or CTAB molecules. Again, the SMILES sequence of citrate, CTAB, and hydroquinone was needed and obtained by PubChem as previously mentioned. To predict target proteins of citrate, CTAB, or hydroquinone, the selected specie was “Homo sapiens” and the SMILES sequence of citrate, CTAB, or hydroquinone was placed at the box “Paste a SMILES in this box, or draw a molecule”, and the “Predict targets” button was pressed. The list of target proteins was presented in a Table.

### 4.10. Instrumentation

The LSPR band of the AuNPs was measured using a Multiskan GO Microplate Spectrophotometer (Thermo Fisher Scientific Inc., Waltham, MA, USA). at 25 °C. The UV–VIS spectra were acquired operating at fast mode and with 1 nm bandwidth. A microplate UV-Star 96-wells from Greiner Bio-One (Kremsmünster, Austria) was used to perform all the UV–VIS measurements (Thermo Fisher Scientific Inc., Waltham, MA, USA).

The surface charge of the nanoparticles was evaluated by zeta potential measurements through electrophoretic light scattering completed in aqueous solutions, using a Zetasizer Nano ZS equipment supplied with a HeNe laser (633 nm) and a scattering detector (173°), from Malvern Panalytical (Malvern, UK). The same equipment was used to assess the hydrodynamic diameter of Au NPs through dynamic light scattering (DLS).

The size and morphology of the AuNPs were investigated by scanning transmission electron microscopy (STEM) using a 200 kV Hitachi HD-2700 STEM microscope (Hitachi High-Technologies Corporation, Tokyo, Japan). AuNPs were prepared for TEM analysis by evaporating suspensions of the nanoparticles on carbon-coated copper grids. The AuNPs were observed as synthesized with exception of gold nanorods, which were centrifuged at 13,300 rpm for 15 min to remove the excess of the stabilizing agent CTAB. The analysis of TEM images to build the histogram of the particle size was performed using the software ImageJ version 1.46.

The incubation of Au NPs in the biofluids was carried out in an orbital shaker incubator (IKA KS 4000 i) at 25 °C at 100 rpm.

The Au concentration in the Au NPs colloids was determined by inductively coupled plasma mass spectrometry (ICP-MS) Thermo X Series (Thermo Fisher Scientific Inc., Waltham, MA, USA). The samples were analyzed as synthesized. The digestion was completed in aqua regia by the addition of 10 μL of HNO_3_ (65%) and 30 μL of HCl (37%) to 500 μL of the colloid. The digestion was completed at RT for periods longer than 48 h.

Savant SpeedVac Vacuum concentrator (Thermo Fisher Scientific Inc., Waltham, MA, USA) was utilized to heat the protein gels until dried and later lyophilized the resulting peptides.

A QExactive Orbitrap with an EASY-spray nanoelectrospray ionization source (Thermo Fisher Scientific), combined to an Ultimate 3000, a high-performance liquid chromatography (HPLC) system (Dionex), was utilized to separate the resulting peptides considering their mass-to-charge ratio. The HPLC system was provided with two columns: a trap column with 100 μm I.D. × 2 cm, packed with Acclaim PepMap RSLC C18 and 5 μm 100 Å; and an EASY-spray analytical with 75 μm I.D. × 15 cm, packed with Acclaim PepMap RSLC C18, 2 μm 100 Å (Thermo Fisher Scientific Inc., Waltham, MA, USA).

## 5. Conclusions

In this study, we investigated the adsorption of biomolecules, especially proteins, on the surface of AuNSs, AuNRs, and AuNFs after incubation with saliva or urine. Monitoring the HD indicated that the adsorption of biomolecules on AuNSs and AuNRs was greatest after 15 and 15–30 min of incubation with saliva and urine, respectively. The AuNFs formed large aggregates after 15 min in contact with saliva, whose size decreased after 60 min. It was suggested that the presence of some proteins as IgG might influence the stability of AuNFs. The LC-MS/MS analysis of AuNSs and AuNRs after 60 min of incubation with saliva led to the identification of 96 proteins. Of the 20 most abundant proteins on the surface of AuNSs and AuNRs, 14 were found in common. The presence of albumin, α-amylase, immunoglobulins heavy constant α, prolactin-inducible protein, and polymeric immunoglobulin receptor can be ascribed to their sulfur content and resultant Au-S interaction. Furthermore, the presence of keratins can be associated to their high molecular weight. The presence of different proteins on the surface of AuNSs and AuNRs was related to the different amount of the common proteins in the first layers of corona, which may contribute to the adsorption of different proteins on the surface of AuNSs and AuNRs. Finally, the different morphology of AuNSs and AuNRs could affect the adsorption of different proteins.

## Figures and Tables

**Table 1:** Wavelength of the LSPR band of Au NPs, before and after the incubation with the biofluids for 15, 30, and 60 min.

	LSPR (nm)						
		Incubation Time (min)					
NPs	As-Prepared	Saliva			Urine		
		15	30	60	15	30	60
AuNSs	549	553	551	553	555	552	547
AuNFs	645	737	748	750	655	655	657
AuNRs	509	518	516	516	514	512	510
	762	670	672	664	724	723	734

## Data Availability

The data presented in this study are available in the present article and in the Supplementary Materials.

## Supplementary Materials

The following Supplementary Materials can be downloaded at: https://www.mdpi.com/article/10.3390/nano12244434/s1, Table S1: Particle dimensions, hydrodynamic diameter (HD) and respective polydispersity index (PDI), zeta potential (ZP), and respective pH of as-synthesized AuNPs, Table S2: PDI of AuNPs before and after being in contact with saliva, Table S3: Protein ID according to Uniprot code and protein respective name identified on the surface of AuNSs and AuNRs, Table S4: The 20 most abundant proteins on the surface of AuNSs and AuNRs and their respective molecular weight (kDa), pI, gravy value, and relative abundance, Table S5: Uniprot code and respective names of the proteins that interact with citrate according to SwissTargetPrediction, Table S6: Molecular weight (kDa), pI, gravy value, and relative abundance of the 18 proteins identified only on the surface of AuNSs or AuNRs, Figure S1: Optical spectra of AuNSs, AuNFs, and AuNRs before and after incubation with saliva and urine for 15, 30, and 60 min, Figure S2: Top—Common proteins on the surface of AuNSs and AuNRs; Bottom—corresponding protein–protein interaction network, and Figure S3: Analysis of PPI network of the 56 proteins common to AuNPs and identified in the Human Salivary Proteome Wiki.
