# Supplementary material for: Interaction of Colloidal Gold Nanoparticles with Urine and Saliva Biofluids: An Exploratory Study
Source: Nanomaterials (Basel). 2022 Dec 13;12(24):4434. doi: 10.3390/nano12244434 (PMC9785464; doi:10.3390/nano12244434)
Supplement: Supplementary file 1 [file nanomaterials-12-04434-s001.zip › nanomaterials-2087790-supplementary.pdf]

Supporting Information

Interaction of Colloidal Gold Nanoparticles with Urine and Saliva Biofluids: An Exploratory Study

Maria António [1], Tânia Lima [2,3], Rui Vitorino [2,4,5,*] and Ana L. Daniel-da-Silva [1,*]

[1] CICECO-Aveiro Institute of Materials, Department of Chemistry, University of Aveiro, 3810-193 Aveiro, Portugal
[2] iBiMED-Institute of Biomedicine, Department of Medical Sciences; University of Aveiro, 3810-193 Aveiro, Portugal
[3] Cancer Biology and Epigenetics Group, Research Center of Portuguese Oncology Institute of Porto (GEBC CI-IPOP) & Porto Comprehensive Cancer Center (P.CCC), 4200-072 Porto, Portugal
[4] LAQV-REQUIMTE, Department of Chemistry, University of Aveiro, 3810-193 Aveiro, Portugal
[5] UnIC@RISE, Department of Surgery and Phycology, Cardiovascular R&D Center, Faculty of Medicine, University of Porto, 4200-319 Porto, Portugal
* Correspondence: rvitorino@ua.pt (R.V.); ana.luisa@ua.pt (A.L.D.-d.-S.)

CONTENTS:

A. Characterization of AuNPs……….....……...…………………………….… 1

B. Protein analysis by LC-MS/MS……………………………….................. 5

A. Characterization of AuNPs

A.1 Physicochemical characterization of Au NPs

Table S1: Particle dimensions, hydrodynamic diameter (HD) and respective polydispersity index (PDI), zeta potential (ZP) and respective pH of as synthesized Au NPs.

	Size (nm)[a]	HD (nm)	PDI	ZP (mV)	pH
**AuNSs**	55.5 ± 8.2	46.3 ± 0.7	0.323 ± 0.002	-61.0 ± 0.3	6.3
**AuNFs**	89.6 ± 15.2	77.9 ± 1.3	0.199 ± 0.004	-58.1± 1.0	3.9
**AuNRs**	20.5 ± 7.3 [b]<br>7.1 ± 1.3 [c]	79.6 ± 6.6	0.192 ± 0.030	71.3 ± 4.9	2.6

a) Assessed by transmission electron microscopy analysis; b) length; c) width

A.1. Optical spectra of AuNPs before and after incubation with biofluids

Figure S1 shows the optical spectra of AuNSs, AuNFs and AuNRs before (as synthesized) and after incubation for 15, 30 and 60 minutes with saliva and urine samples.

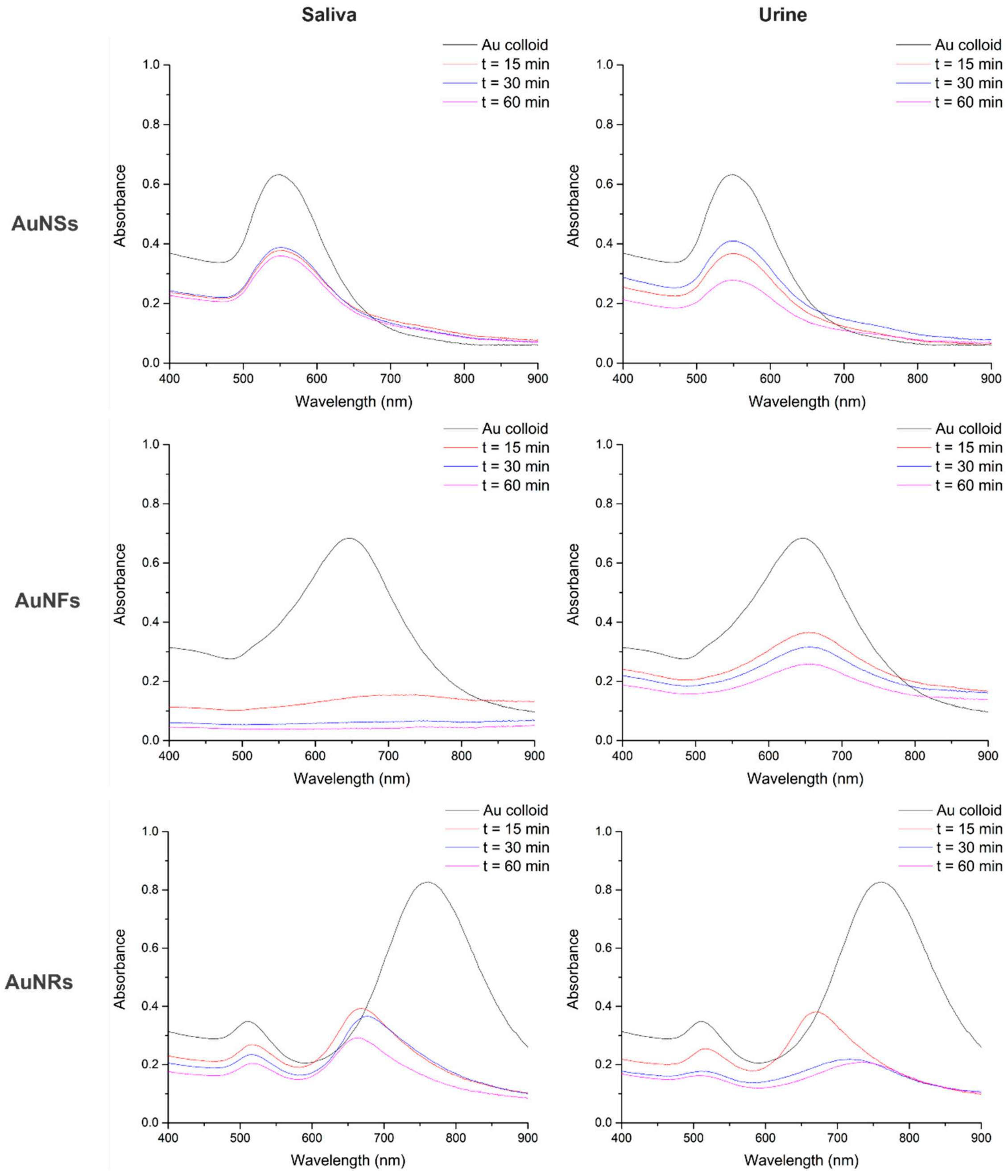

Figure S1: Optical spectra of AuNSs, AuNFs and AuNRs before and after incubation with saliva and urine for 15, 30 and 60 minutes.

A.2. **Zeta potential and hydrodynamic diameter AuNPs**

The AuNFs demonstrate a high aggregation tendency over time when incubated with saliva, consequently, they were characterized through polydisperse index (PDI), as table S1 presents.

Table S2: PDI of AuNPs before and after being in contact with saliva.

Sample	Time (minutes)	PDI
AuNFs	0	0.199 ± 0.015
	15	0.886 ± 0.047
	30	1 ± 0.001
	60	0.586 ± 0.049

B. Protein analysis by LC-MS/MS

Table S3: Protein ID according to Uniprot code and protein respective name identified on the surface of AuNSs and AuNRs.

Protein ID	Name
**P15515**	Histatin-1
**P05109**	Protein S100-A8
**P01040**	Cystatin-A
**P04080**	Cystatin-B
**P0DOY2**	Immunoglobulin lambda constant 2
**P01834**	Immunoglobulin kappa constant
**P01700**	Immunoglobulin lambda variable 1-47
**P01619**	Immunoglobulin kappa variable 3-20
**P01624**	Immunoglobulin kappa variable 3-15
**A0A0A0MRZ8**	Immunoglobulin kappa variable 3D-11
**P01764**	Immunoglobulin heavy variable 3-23
**A0A0C4DH38**	Immunoglobulin heavy variable 5-51
**A0A0B4J1X5**	Immunoglobulin heavy variable 3-74
**P01599**	Immunoglobulin kappa variable 1-17
**A0A0C4DH31**	Immunoglobulin heavy variable 1-18
**P01780**	Immunoglobulin heavy variable 3-7
**A0A0C4DH68**	Immunoglobulin kappa variable 2-24
**P06702**	Protein S100-A9
**A0A0B4J1Y9**	Immunoglobulin heavy variable 3-72
**P06310**	Immunoglobulin kappa variable 2-30
**P01614**	Immunoglobulin kappa variable 2D-40
**P01824**	Immunoglobulin heavy variable 4-39
**P69905**	Hemoglobin subunit alpha
**P01034**	Cystatin-C
**P02766**	Transthyretin
**P68871**	Hemoglobin subunit beta
**P28325**	Cystatin-D
**P01036**	Cystatin-S
**P09228**	Cystatin-SA
**P01037**	Cystatin-SN
**P61626**	lysozyme c
**P12273**	Prolactin-inducible protein
**P01591**	Immunoglobulin J chain
**P80188**	Neutrophil gelatinase-associated lipocalin
**Q6MZM9**	Proline-rich protein 27
**Q96DA0**	Zymogen granule protein 16 homolog B
**P0DOX8**	Immunoglobulin lambda-1 light chain
**P01033**	Metalloproteinase inhibitor 1
**P09211**	Glutathione S-transferase P
**P0DOX7**	Immunoglobulin kappa light chain
**Q96DR5**	BPI fold-containing family A member 2

**P06870**	Kallikrein-1
**P54108**	Isoform 3 of Cysteine-rich secretory protein 3
**P25311**	Zinc-alpha-2-glycoprotein
**P23280**	Carbonic anhydrase 6
**P01859**	Immunoglobulin heavy constant gamma 2
**P04406**	Glyceraldehyde-3-phosphate dehydrogenase
**P01876**	Immunoglobulin heavy constant alpha 1
**P07858**	Cathepsin B
**Q6P5S2**	Protein LEG1 homolog
**Q8TAX7**	Mucin-7
**P60709**	Actin, cytoplasmic 1
**P07339**	Cathepsin D
**P29508**	Serpin B3
**P01009**	Alpha-1-antitrypsin
**P06733**	Alpha-enolase
**Q04695**	Keratin, type I cytoskeletal 17
**P20061**	Transcobalamin-1
**P0DOX2**	Immunoglobulin alpha-2 heavy chain
**Q8N4F0**	BPI fold-containing family B member 2
**P0DOX5**	Immunoglobulin gamma-1 heavy chain
**P13646**	Keratin, type I cytoskeletal 13
**P80303**	Nucleobindin-2
**P08779**	Keratin, type I cytoskeletal 16
**P02533**	Keratin, type I cytoskeletal 14
**Q02818**	Nucleobindin-1
**P02774**	Isoform 3 of Vitamin D-binding protein
**Q14CN4**	Keratin, type II cytoskeletal 72
**P04745**	Alpha-amylase 1
**P10909**	Isoform 2 of Clusterin
**P07602**	Isoform Sap-mu-9 of Prosaposin
**P13645**	Keratin, type I cytoskeletal 10
**P48668**	Keratin, type II cytoskeletal 6C
**Q7Z794**	Keratin, type II cytoskeletal 1b
**P35527**	Keratin, type I cytoskeletal 9
**P13647**	Keratin, type II cytoskeletal 5
**Q5T749**	Keratinocyte proline-rich protein
**Q08380**	Galectin-3-binding protein
**P35908**	Keratin, type II cytoskeletal 2 epidermal
**P04264**	Keratin, type II cytoskeletal 1
**P02768**	Serum albumin
**Q14515**	SPARC-like protein 1
**P02787**	Serotransferrin
**P02788**	Lactotransferrin
**P22079**	Lactoperoxidase
**P14923**	Junction plakoglobin
**P01833**	Polymeric immunoglobulin receptor

**P09958**	Furin
**P05164**	Isoform H7 of Myeloperoxidase
**P32926**	Desmoglein-3
**Q02413**	Desmoglein-1
**P01024**	Complement C3
**Q9UGM3**	Deleted in malignant brain tumors 1 protein
**P15924**	Desmoplakin
**Q9Y6R7**	IgGFc-binding protein
**Q9HC84**	Mucin-5B

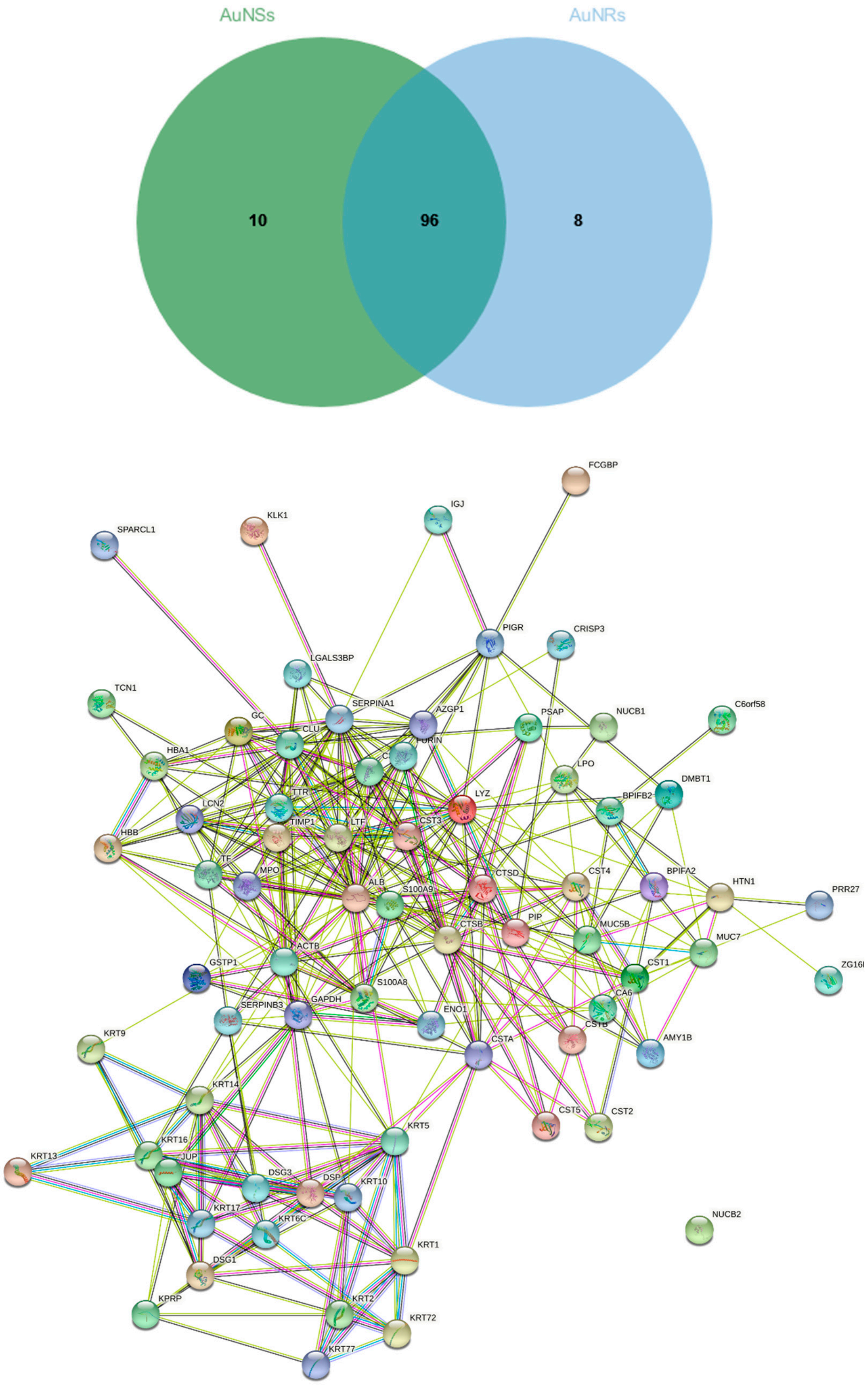

Figure S2: Top – Common proteins on the surface of AuNSs and AuNRs; Bottom – Corresponding protein-protein interaction network.

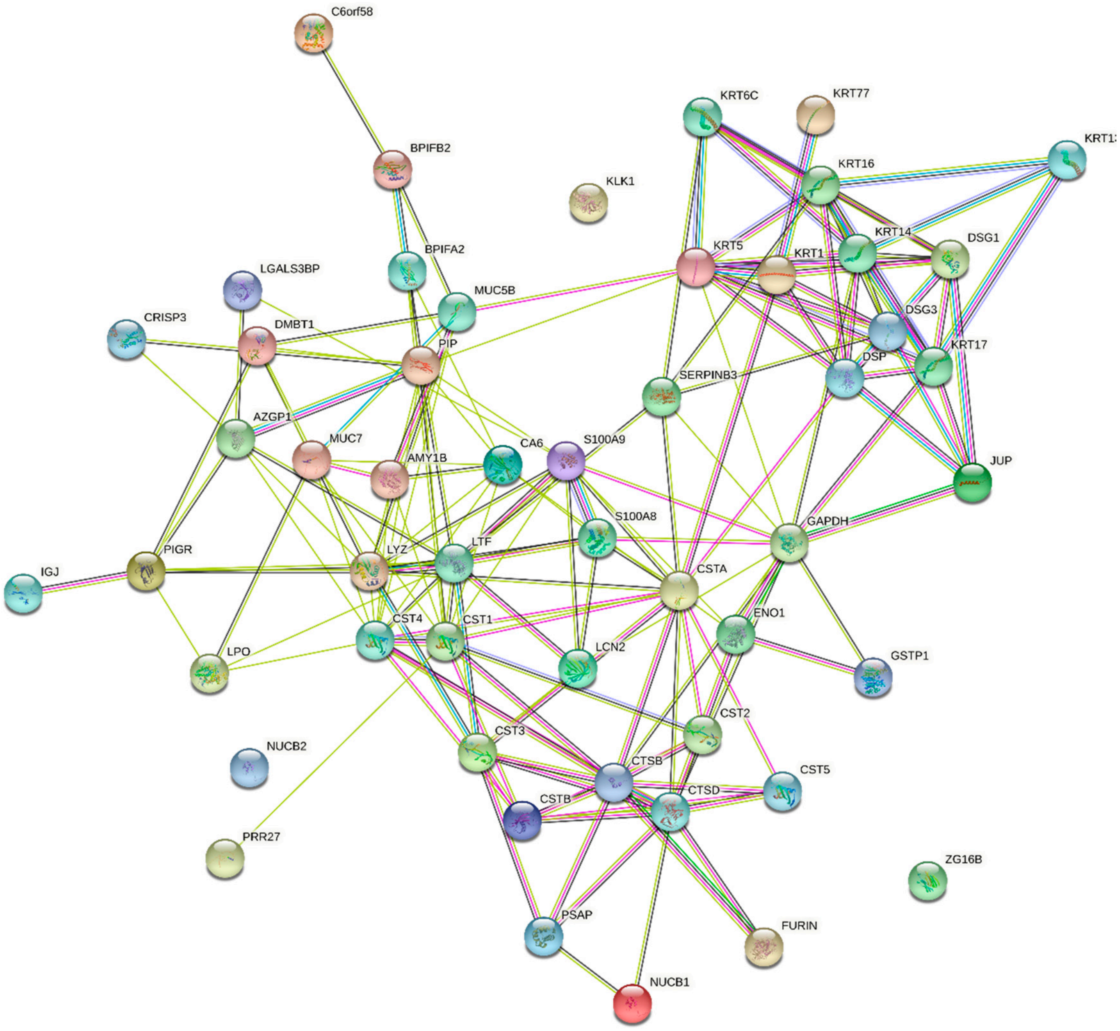

Figure S3: Analysis of PPI network of the 56 proteins common to AuNPs and identified in the Human Salivary Proteome Wiki.

Table S4: The 20 most abundant proteins on the surface of AuNSs and AuNRs and their respective molecular weight (kDa), pI, gravy value and relative abundance.

Protein ID	Name	MW (kDa)	pI	Gravy value	Abundance (10^8)
AuNSs					
**P01036**	Cystatin-S	16.2	5.02	-0.399	9.03
**P01876**	Immunoglobulin heavy constant alpha 1	37.6	6.51	-0.206	6.27
**P04745**	Alpha-amylase 1	57.7	6.93	-0.435	4.45
**P04264**	Keratin, type II cytoskeletal 1	66	8.12	-0.626	3.29
**P01833**	Polymeric immunoglobulin receptor	83.2	5.74	-0.341	2.23
**P01037**	Cystatin-SN	16.4	7.21	-0.440	2.16
**P13645**	Keratin, type I cytoskeletal 10	58.8	5.21	-0.623	2.04
**P02768**	Serum albumin	69.3	6.28	-0.353	1.93
**P35527**	Keratin, type I cytoskeletal 9	62	5.24	-0.700	1.40
**P61626**	Lysozyme c	16.5	9.16	-0.195	1.26
**P35908**	Keratin, type II cytoskeletal 2 epidermal	65.4	8	-0.471	1.24
**P01834**	Immunoglobulin kappa constant	11.8	6.52	-0.537	1.24
**Q96DA0**	Zymogen granule protein 16 homolog B	22.7	7.39	-0.163	0.776
**P12273**	Prolactin-inducible protein	16.6	8.05	-0.175	0.589
**P01034**	Cystatin-C	15.8	8.75	-0.111	0.493
**P01591**	Immunoglobulin J chain	18.1	5.24	-0.338	0.419
**P0DOX8**	Immunoglobulin lambda-1 light chain	22.8	6.76	-0.447	0.367
**P0DOX2**	Immunoglobulin alpha-2 heavy chain	48.9	6.81	-0.235	0.302

**P28325**	Cystatin-D	16.1	7.17	-0.242	0.214
**P02533**	Keratin, type I cytoskeletal 14	51.5	5.16	-0.537	0.158
AuNRs					
**P04264**	Keratin, type II cytoskeletal 1	66	8.12	-0.626	4.37
**P13645**	Keratin, type I cytoskeletal 10	58.8	5.21	-0.623	2.93
**P04745**	Alpha-amylase 1	57.7	6.93	-0.435	2.60
**P35527**	Keratin, type I cytoskeletal 9	62	5.24	-0.700	2.11
**P01036**	Cystatin-S	16.2	5.02	-0.399	1.64
**P02768**	Serum albumin	69.3	6.28	-0.358	1.62
**P35908**	Keratin, type II cytoskeletal 2 epidermal	65.4	8	-0.471	1.44
**P01876**	Immunoglobulin heavy constant alpha 1	37.6	6.51	-0.206	0.787
**P01037**	Cystatin-SN	16.4	7.21	-0.440	0.403
**P23280**	Carbonic anhydrase 6	35.3	7.02	-0.465	0.388
**P06870**	Kallikrein-1	28.9	4.83	-0.113	0.332
**P25311**	Zinc-alpha-2-glycoprotein	34.2	6.05	-0.576	0.321
**P12273**	Prolactin-inducible protein	16.6	8.05	0.175	0.310
**P01833**	Polymeric immunoglobulin receptor	83.2	5.74	-0.341	0.279
**P02533**	Keratin, type I cytoskeletal 14	51.5	5.16	-0.537	0.267
**Q96DA0**	Zymogen granule protein 16 homolog B	22.7	7.39	-0.163	0.224
**P01834**	Immunoglobulin kappa constant	11.8	6.52	-0.537	0.213
**Q8TAX7**	Mucin-7	39.1	8.78	-0.597	0.172
**P13647**	Keratin, type II cytoskeletal 5	62.3	7.74	-0.428	0.126
**P48668**	Keratin, type II cytoskeletal 6C	60	8	-0.432	0.114

Table S5: Uniprot code and respective names of the proteins that interact with citrate according to SwissTargetPrediction.

Protein ID	Name
**P53396**	ATP-citrate synthase
**P37268**	Squalene synthetase *(by homology)*
**P36544**	Neuronal acetylcholine receptor protein alpha-7 subunit *(by homology)*
**Q9GZT9**	Eglnine homolog 1
**P04035**	HMG-CoA reductase
**Q4U2R8**	Solute carrier family 22 member 6 *(by homology)*
**P49354/P49356**	Protein farnesyltransferase
**P43005**	Excitatory amino acid transporter 3
**P43116**	Prostanoid EP2 receptor
**P43088**	Prostanoid FP receptor
**Q9UHC9**	Niemann-Pick C1-like protein 1
**Q9UJM8**	Hydroxyacid oxidase 1
**Q9Y2K7**	Lysine-specific demethylase 2A
**Q9UPP1**	Histone lysine demethylase PHF8
**P41229**	Lysine-specific demethylase 5C
**Q14832**	Metabotropic glutamate receptor 3
**Q14416**	Metabotropic glutamate receptor 2
**P39086**	Glutamate receptor ionotropic kainate 1
**P42261**	Glutamate receptor ionotropic, AMPA 1
**P0DMS8**	Adenosine A3 receptor
**Q16478**	Glutamate receptor ionotropic kainate 5
**Q14833**	Metabotropic glutamate receptor 4
**P48058**	Glutamate receptor ionotropic, AMPA 4
**P41594**	Metabotropic glutamate receptor 5
**O00222**	Metabotropic glutamate receptor 8
**Q13002**	Glutamate receptor ionotropic kainate 2
**Q13003**	Glutamate receptor ionotropic kainate 3
**Q13255**	Metabotropic glutamate receptor 1
**Q14831**	Metabotropic glutamate receptor 7

**P42262**	Glutamate receptor ionotropic, AMPA 2
**O15303**	Metabotropic glutamate receptor 6
**P16662**	UDP-glucuronosyltransferase 2B7
**Q04609**	Glutamate carboxypeptidase II
**P52209**	6-phosphogluconate dehydrogenase
**P15090**	Fatty acid binding protein adipocyte
**Q07869**	Peroxisome proliferator-activated receptor alpha
**P05413**	Fatty acid binding protein muscle
**Q01469**	Fatty acid binding protein epidermal
**Q03181**	Peroxisome proliferator-activated receptor delta
**O14842**	Free fatty acid receptor 1
**P12104**	Fatty acid binding protein intestinal
**Q96RI1**	Bile acid receptor FXR
**P11413**	Glucose-6-phosphate 1-dehydrogenase
**Q8TDU6**	G-protein coupled bile acid receptor 1
**P50416**	Carnitine O-palmitoyltransferase 1, liver isoform *(by homology)*
**P28845**	11-beta-hydroxysteroid dehydrogenase 1
**P80365**	11-beta-hydroxysteroid dehydrogenase 2
**P10275**	Androgen Receptor
**P11473**	Vitamin D receptor
**P11511**	Cytochrome P450 19A1
**P47869/P47870 /P18507**	GABA A receptor alpha-2/beta-2/gamma-2
**P30304**	Dual specificity phosphatase Cdc25A
**O60218**	Aldo-keto reductase family 1 member B10
**P06746**	DNA polymerase beta *(by homology)*
**O15379**	Histone deacetylase 3
**P43004**	Excitatory amino acid transporter 2
**P00915**	Carbonic anhydrase I
**Q16790**	Carbonic anhydrase IX
**Q9H4A4**	Aminopeptidase B
**O14684**	Prostaglandin E synthase
**P08185**	Corticosteroid binding globulin
**P04278**	Testis-specific androgen-binding protein

**P37058**	Estradiol 17-beta-dehydrogenase 3
**Q9UBS5**	GABA-B receptor *(by homology)*

Table S6: Molecular weight (kDa), pI, gravy value and relative abundance of the 18 proteins identified only on the surface of AuNSs or AuNRs.

AuNPs	Protein ID	Name	MW (kDa)	pI	Gravy value	Abundance (10^5)
AuNSs	P16870	Carboxypeptidase E	53.1	5.14	-0.449	2.05
	P11021	Endoplasmatic reticulum chaperone BiP	72.3	5.16	-0.486	0.458
	P02741	C-reactive protein	25	5.63	-0.0321	20.6
	P00450	Ceruloplasmin	122.1	5.72	-0.536	0.0689
	A8K2U0	Alpha-2-macroglobulin-like protein 1	161	5.73	-0.157	0.620
	P01871	Immunoglobulin heavy constant mu	51.9	6.15	-0.319	33.1
	P04792	Heat shock protein beta-1	22.8	6.4	-0.567	2.11
	P01782	Immunoglobulin heavy variable 3-9	12.9	7.08	-0.0271	1.37
	P04259	Keratin, type II cytoskeletal 6B	60	8	-0.451	0.390
	A0A0C4DH34	Immunoglobulin heavy variable 4-28	13.1	9.29	-0.00513	0.156
AuNRs	Q8NBJ4	Golgi membrane protein 1	45.3	4.97	-0.983	0.178
	Q8NFT8	Delta and Notch-like epidermal growth factor-related receptor	78.4	5.17	-0.151	0.0999
	Q6S8J3	POTE ankyrin domain family member E	121.3	6.2	-0.682	1.85
	P30740	Leukocyte elastase inhibitor	42.7	6.28	-0.248	2.15
	P00738	Haptoglobin	45.2	6.58	-0.421	1.86
	P04083	Annexin A1	38.7	7.02	-0.419	0.0756
	P02790	Hemopexin	51.6	7.02	-0.434	0.189
	O00584	Ribonuclease T2	29.5	7.08	-0.582	0.451
